# Reference Charts for Neonatal Cranial Volume Based on 3D Laser Scanning to Monitor Head Growth

**DOI:** 10.3389/fped.2021.654112

**Published:** 2021-05-28

**Authors:** Marijn Jorien Vermeulen, Wolfram Burkhardt, Anne Fritze, Jorine Roelants, Lars Mense, Sten Willemsen, Mario Rüdiger

**Affiliations:** ^1^Division of Neonatology, Department of Pediatrics, Erasmus MC University Medical Center, Rotterdam, Netherlands; ^2^Division of Neonatology and Pediatric Intensive Care Medicine, Department of Pediatrics, Medizinische Fakultät der Technischen Universität, Dresden, Germany; ^3^Division of Biostatistics, Erasmus MC University Medical Center, Rotterdam, Netherlands; ^4^Saxonian Center for Feto-Neonatal Health, Technische Universität Dresden, Dresden, Germany

**Keywords:** cranial volume, head circumference, head volume, reference chart, percentile, brain growth, gender, preterm

## Abstract

**Background:** Postnatal brain growth is an important predictor of neurodevelopmental outcome in preterm infants. A new reliable proxy for brain volume is cranial volume, which can be measured routinely by 3-D laser scanning. The aim of this study was to develop reference charts for normal cranial volume in newborn infants at different gestational ages starting from late preterm for both sexes.

**Methods:** Cross-sectional cohort study in a German university hospital, including singleton, clinically stable, neonates born after 34 weeks of gestation. Cranial volume was measured in the first week of life by a validated 3-D laser scanner. Cranial volume data was modeled to calculate percentile values by gestational age and birth weight and to develop cranial volume reference charts for girls and boys separately.

**Results:** Of the 1,703 included infants, 846 (50%) were female. Birth weights ranged from 1,370 to 4,830 grams (median 3,370). Median cranial volume ranged from 320 [interquartile range (IQR) 294–347] ml at 34 weeks to 469 [IQR 442–496] ml at 42 weeks and was higher in boys than in girls.

**Conclusions:** This study presents the first reference charts of cranial volume which can be used in clinical practice to monitor brain growth between 34 and 42 weeks gestation in infants.

## Introduction

Very preterm birth is associated with poor brain growth and reduced brain volume throughout childhood and adolescence (1–3). Not only in the window of preterm, but also of term birth, gestational age at birth is associated with brain size in later childhood (4). Small brain size in the neonatal period is related to impaired neurodevelopment and academic performance, which obviously can impact quality of life (1, 5, 6). In preterm infants, brain growth may be hampered by many factors associated with preterm birth itself, such as inflammation, hypoxaemia or hyperoxaemia and intraventricular hemorrhage (3). However, brain growth is also affected by modifiable factors in neonatal care such as high dose corticosteroids, painful procedures and inadequate nutritional intake (7–9). As nutritional requirements change over time and may even differ between girls and boys, (10) brain growth monitoring can be a useful tool in individualization of nutritional treatment.

Currently, monitoring of brain growth is usually based on the widespread method of repeated tape measurement of head circumference. However, this approach measures head circumference as 2-dimensional (2-D) and does not always adequately reflect head and brain volume, especially in infants at lower gestational age (11, 12). The most reliable method to measure brain volume and growth is cranial magnetic resonance imaging (MRI) (13). However, MRI is not appropriate for routine monitoring because repeated measurements are prohibitively expensive, technically demanding and not available in all clinical settings. 3-D Ultrasound techniques are being developed, but are not yet suitable for measuring neonatal cranial volumes (14).

Digital 3-D laser scanning of the head represents a safe, non-invasive and technically sound alternative for measuring an infant's cranial volume with high reproducibility and inter-observer agreement (12). We have recently shown that cranial volume as assessed by 3-D laser scanning is a reliable proxy for brain volume as compared to cranial MRI (15). Since 3-D laser scanning can be performed repeatedly, cranial volume could become an important parameter in routine neonatal growth monitoring, especially useful in preterm infants with high risk of impaired brain growth. To target interventions on optimizing brain growth in very preterm infants, normal values of neonatal cranial volumes are needed. These should be based on birth data of healthy late preterm and term infants, as in that age range growth is a main focus of neonatal care. Therefore, the aim of this study was to develop reference charts and tables for cranial volumes across a relevant range of gestations, which can be used for individual monitoring of neonatal head growth.

## Methods

### Study Setting and Population

We developed references values for cranial volume based on measurements shortly after birth, in a cross-sectional cohort study at the tertiary teaching hospital in Dresden, Germany. Cranial volume measurement has been introduced into clinical routine for all neonates admitted to the intermediate care or maternity ward since 2010 and is performed three times a week.

We included neonates, who were admitted to either the maternity ward or intermediate care unit, between April 2011 and November 2014, who were scanned in their first week of life. The sample size was determined by convenience and expected to provide sufficient data at the tails of the distribution to model growth charts. Digital head scanning was done only in infants without obvious head deformities such as cephalohaematoma, hydrocephalus or skull anomalies. As measurement is only possible without (respiratory) devices attached to the head, infants admitted to the intensive care unit were not included. Due to small numbers, we excluded infants born before 34 0/7 weeks of gestation. To select a sample most representative of physiological fetal growth in the general population, we excluded multiple births such as twins or triplets, as well as infants being very small or large for gestational age with a birth weight below or above 2 standard deviations (SD) on Fenton‘s growth reference chart (16). We also excluded extreme outliers with a cranial volume values outside the 4 SD range for gestational age or birth weight, based on a basic preliminary model.

### 3-D Cranial Volume Measurement

Cranial volume was measured by a medical assistant using a non-invasive 3-D laser shape digitizer (STARscanner™, Orthomerica Products Inc, Orlando, FL, USA), as described previously in full detail (12). In brief, after placing the infant in the scanner, a 3-D head shape was captured within a few seconds, by four Class-I eye-safe lasers and eight cameras. Using specialized software (YETI™ Shape Builder and Head Comparison Utility, Vorum Research Corporation, Vancouver, Canada) cranial volume and head circumference were calculated based on predefined anatomical reference planes (12).

### Data Collection and Statistical Analyses

Descriptive data were obtained from patient records and included sex, gestational age, weight, length and head circumference at birth and postnatal age at measurement. If not noted otherwise, medians [1^st^-3^rd^ quartile] are reported. All variables for the analyses were complete. Comparisons of characteristics and head volume between boys and girls was by use of *T*-tests and linear regression analyses (SPSS, version 21 for Windows, IBM, United States of America). *P*-values below 0.05 were considered statistically significant.

Models were built for cranial volume as a function of gestational age and birth weight for boys and girls separately, using Generalized Additive Models for Location, Scale and Shape in R (GAMLSS) (17). For each of the 4 models, we first evaluated the goodness of fit with two different distributions: the normal and Box, Cox, Cole and Green. Then, we selected the model best fitting the observations by comparing the Generalized Akaike information criterion with a penalty of 3 per parameter.

The model by gestational age used a normal distribution with constant variance and a spline with approximately 3 (SD 2) effective degrees of freedom for the mean for boys and girls. The model by birth weight also used a normal distribution with constant variance and a spline with approximately 4 (SD 2) effective degrees of freedom for boys and 3 for girls. Box, Cox Cole and Green models, allowing for deviations from normality, did not improve any of the models. With the selected distributions, all models provided adequate goodness-of-fit. Quantiles of cranial volume and tail probabilities are reported by gestational age and birth weight, for different percentiles.

In all cases the normal model was preferred over the Box-Cox-Cole and Green model. The effective degrees of freedom for the mean cranial volume as a function of gestational birthweight was ~4. For this reference chart the log standard deviation was linear. For all other reference curves, the effective degrees of freedom were ~3 for the mean and the standard deviation was constant. With the selected distributions, all models provided adequate goodness-of-fit. Quantiles of cranial volume and tail probabilities are reported by gestational age and birth weight, for different percentiles.

Based on the growth models, percentile values and reference charts for cranial volume were formulated by gestational age and birth weight for both sexes using the gamlss package (version 5.1) by Rigby and Stasinopoulos for R (using version 3.4.1, 2017 for Windows, R Foundation for Statistical Computing, Vienna, Austria). The precision of the quantiles was estimated using boot-strapping to estimate 95% confidence intervals for the 10^th^, 50^th^, and 90^th^ percentiles. Based on the confidence intervals, the x-axes of the curves were truncated to a reasonable range.

With no longitudinal data available, growth rates were extrapolated from the modeled cross-sectional data. By calculating relative differences in median cranial volume over time (per week of gestational age), growth rates were estimated for the hypothetical case that tracks the 50^th^ percentile.

### Ethics Statement

The ethics committee of the Medical Faculty Carl Gustav Carus of the Technical University Dresden, Germany regarded the study as an audit of standard care and decided that no specific written parental consent was needed, as all neonates underwent cranial volume measurements as part of standard care with approved devices, and data collection was anonymized (EK2610821012).

## Results

Of 1,893 neonates with cranial volume measured, 22 were excluded because of a gestational age below 34 weeks, 45 and 17 neonates being small and large for gestational age respectively, 70 twins, 35 neonates with first cranial volume measurement after day 7 and 1 outliers with a cranial volume value above +4 SD (Supplemental Figure 1). Of the 1,703 (90%) neonates included, 846 (50%) were female. Gestational age ranged from 34^0/7^ to 42^2/7^ weeks (median 39^4/7^ [IQR 38^4/7^-40^4/7^]), birth weight from 1370 to 4830 g (3370 [3010–3670]) and head circumference from 28 to 39 cm (35 [34–36]). In the majority (91%) of neonates, cranial volume was measured within 3 days after birth. All cranial volume measurements were successful. For the total group, median cranial volume was 428 ml [393–460]. The median ranged from 320 ml [294–347] at 34 weeks to 469 [442–496] ml at 42 weeks of gestation.

Though mean gestational age at birth was not different, mean weight, length and head circumference were higher in boys than in girls (Table 1). Mean cranial volume was larger in boys than in girls [mean difference 27 ml (95%-confidence interval 23–32)], a difference that remained significant even after adjusting for gestational age, birth weight and postnatal day at measurement (*p* < 0.001).

**Table 1:** Neonatal characteristics.

	**Male (*n* = 857)**	**Female (*n* = 846)**	***P***
Gestational age, weeks	39^4/7^ (38^5/7^;40^4/7^)	39^4/7^ (38^4/7^;40^4/7^)	0.52
Birth weight, g	3,422 (508)	3,239 (512)	<0.0001
Birth weight, Z-score	0.0 (0.8)	−0.1 (0.8)	0.05
Birth length, cm^1^	51.0 (2.4)	50.3 (2.5)	<0.0001
Birth head circumference, cm^2^	35.4 (1.6)	34.7 (1.6)	<0.0001
Age at measurement, days	1.9 (1.3)	1.9 (1.4)	0.43
Cranial volume, ml	439 (50)	412 (48)	<0.0001

*Data are presented as mean (SD) and mean difference (95% confidence interval) with p-value for two sample T-test, except for gestational age (being not normally distributed) that is presented as median (interquartile range) with p-value for Mann-Whitney U Test and preterm (<37 weeks) presented as number (%) with p-value for Chi-square test. ^1^ 1 missing (0.1%), ^2^ 13 missing (0.8%)*.

**Figure 1:**
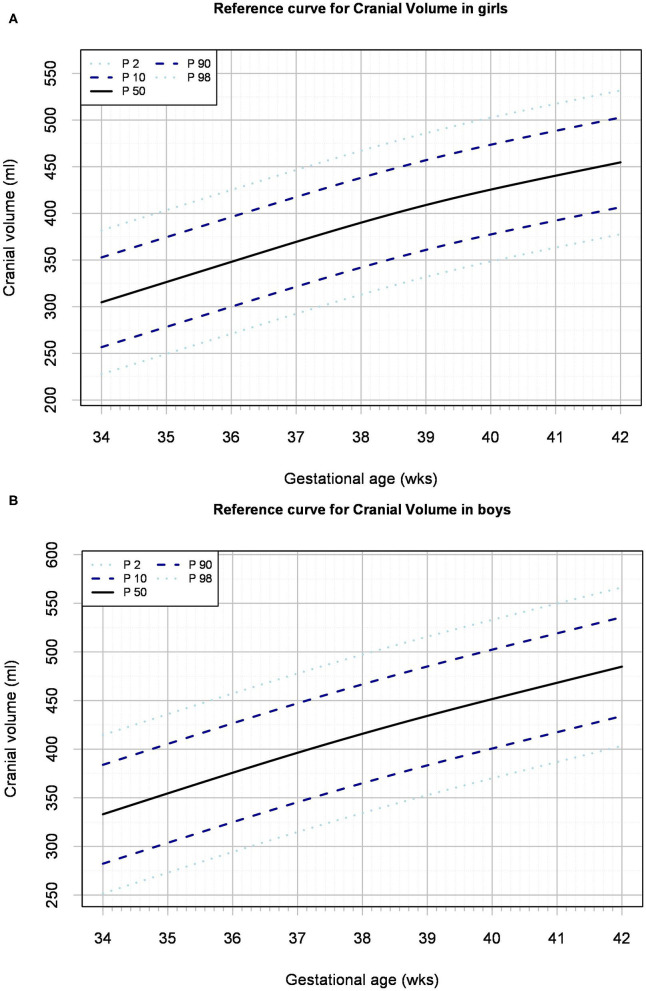
Reference curve for cranial volume by gestational age for girls **(A)** and boys **(B)**.

**Figure 2:**
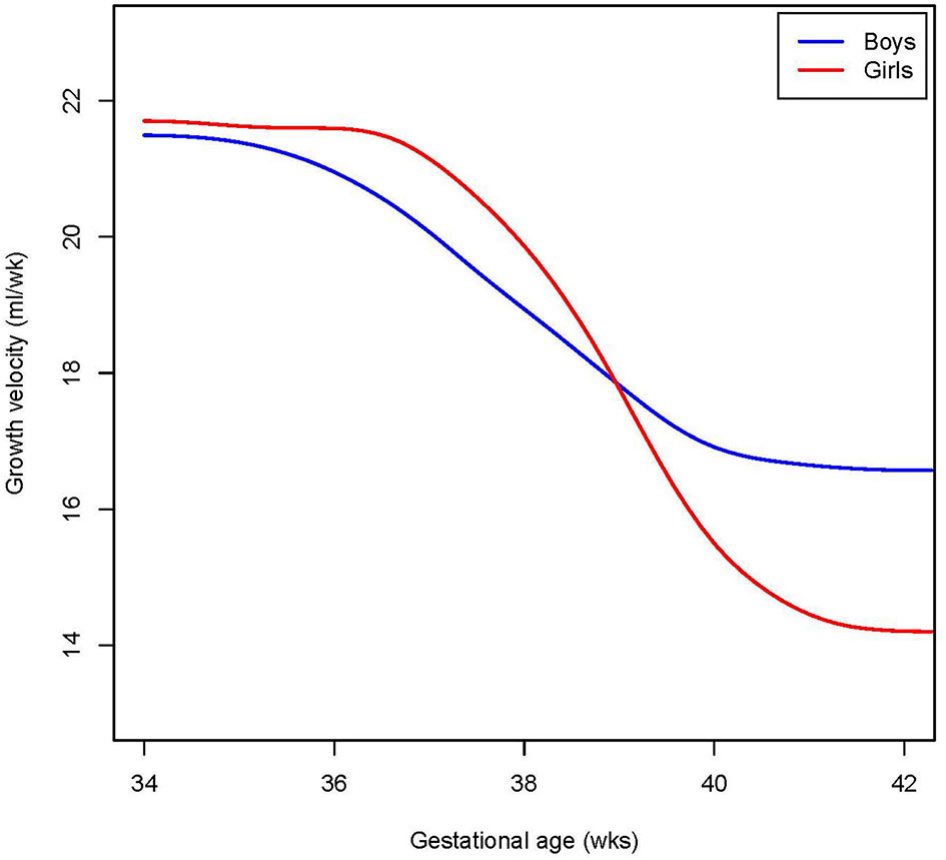
Cranial volume growth rate. Shown is the extrapolated median growth rate per week by gestational age, in girls and boys for those tracking the mean.

Reference charts for cranial volume are shown by gestational age (Figure 1) and birth weight (Supplementary Figure 2) for both sexes, with absolute values and distribution parameters presented in Supplementary Tables 1 and 2, respectively. The distribution of the raw data underlying the models, and the 95% confidence intervals, are shown in Supplementary Figures 3A–D and 4A–D, respectively. Based on the median cranial volume values in the reference charts, fetal growth rate was estimated, which declined from 21 to 17 ml/week in boys and from 22 to 14 ml/week in girls between 34 and 42 weeks (Figure 2).

## Discussion

This study provides the first reference charts of cranial volume in late preterm and term neonates. The charts may be used to plot individual cranial volume values to monitor individual head growth in preterm infants. In addition to cranial volume charts by gestational age, we provide charts relative to birth weight. These last charts can be useful to quantify disproportional head growth, as seen in “brain sparing” due to insufficient nutritional intake.

Our reference values include data from 34 weeks of gestation until term, that are relevant for preterm infants. After extreme or very preterm birth, this period refers to the more stable stage of recovery after critical illness, in which growth and neurodevelopment are the main focus of care. Our reference values do not include preterm infants born below 34 weeks of gestation, because the current device does not allow for scanning during respiratory support.

Cranial volume was higher in boys than in girls at equal gestational ages. This finding is consistent with fetal and birth cohort studies showing that in general, head circumference (by fetal ultrasound and postnatal tape measurements) and brain volume (on MRI) are larger in boys than in girls (10, 16, 18). Although prospective longitudinal volume data are currently lacking, sex differences likely relate to different development of brain structures, as well as to different nutritional needs (13, 18, 19).

As compared to measuring simple head circumference, a 3-D approach has several advantages. First of all, we earlier showed that it provides a more reliable proxy for brain volume than 2-D measurements (15). Second, there is less measurement error due to head deformities. These are commonly seen in preterm infants, due to attached medical devices (such as CPAP caps) and head positioning in nursing care in combination with low muscle tone and soft cranial bone structures (20).

Our data are based on the STARscanner^TM^, which is easy to use and quickly provides cranial volume data at high accuracy and reproducibility (12). Development of more sophisticated 3-D devices is ongoing. We expect cheaper and more accessible tools in the future that will become useful for brain growth monitoring pending they meet the following criteria: (i) easy to use in a clinical setting, including applicability in infants in incubators on respiratory support; (ii) short picture acquisition time in order to minimize motion artifacts; (iii) low acquisition and operating costs; (iv) calculation of cranial volume based on validated algorithms to predict brain volume, for example, using automatic measurement based on standard anatomical points (12).

A strength of the present study is the large sample of healthy neonates in a wide range of gestational ages and birth weights. Our data are unique, as earlier reports on brain volumes are MRI-based, describe smaller samples and lack sex-specific reference values and charts (13). As cranial volume determined by 3-D laser scanning is strongly correlated to MRI-based brain volume, the reference charts may also be used as a proxy for brain volumes measured by other 3-D techniques (15).

Limitations of our study include the uneven distribution of collected data, with lower numbers at lower gestational age, caused by the availability of the routinely collected data. This explains the larger confidence intervals at the tails of the curves, underlining the need for cautious interpretation of the percentile lines at low gestational age (21). The convenience dataset reflects the usual distribution among different gestational ages and was large enough to create models that fitted the data well. This is supported by the 95% confidence intervals for the boys' curves, ranging around 10–40 ml for the gestational age and birth weight curves. However, the confidence intervals for the percentiles for girls were much larger. Specifically these models would benefit of a larger sample of girls at lower gestational ages. Therefore, we strongly encourage further study and validation of our models in other cohorts. Inspired by the step-wise development of neonatal growth charts by the WHO, we look forward to update the cranial volume charts, by meta-analyses of future cohort studies (16).

Interpretation and clinical use of growth reference values always requires critical appraisal of the reference population. Selection of a sample representative of the general population was limited by our tertiary hospital population, single-center design and predominantly Caucasian population. As we aimed to provide prescriptive (or: normative) charts that reflect general physiological growth, we excluded a few groups on clinical grounds. We excluded twins and severely growth restricted neonates, because we expected higher cranial volume in relation to their birth weight by fetal brain-sparing. Also, neonates with severe macrosomia were excluded, as they are likely to have small cranial volumes relative to their birth weight by body fat accumulation. By selecting on birth weight and not on cranial volume, we expect to have captured sufficient general biological variation in cranial volume in our data.

We provide cross-sectional data measured soon after birth, that represent the postnatal result of fetal head growth. Postnatal growth after preterm birth mostly differs from fetal growth, for example due to critically illness, nutritional deficits, and use of steroids. Although a positive correlation between cranial volume and subsequent neurodevelopmental outcome is supported by previous studies, (22, 23) strong evidence is lacking to what extend targeting at fetal growth postnatally is most optimal. In our center, investigations are continuing on a new cohort of very preterm born infants, providing longitudinal cranial volume data for the first year of life. By relating cranial growth trajectories to neurodevelopmental outcome, we hope to define optimal postnatal cranial volume growth trajectories.

In conclusion, earlier studies showed that 3-D laser scanning is an easy and accurate method to measure neonatal cranial volume. We now provide reference charts and tables to be used for cranial volume monitoring during routine neonatal care. These reference values may be used to support development of new clinical interventions, such as personalized nutritional care, to improve brain growth and later outcome after preterm birth.

## Data Availability Statement

The datasets generated for this article are not readily available because of legal limitations (institutional and national legislation). In specific cases, the raw data supporting the conclusions of this article can be made available by the authors, upon individual request. Requests to access the datasets should be directed to m.j.vermeulen@erasmusmc.nl.

## Ethics Statement

The studies involving human participants were reviewed and approved by The ethics committee of the Medical Faculty Carl Gustav Carus of the Technical University Dresden, Germany (EK2610821012). Written informed consent from the participants' legal guardian/next of kin was not required to participate in this study in accordance with the national legislation and the institutional requirements.

## Author Contributions

MV and JR conceptualized and designed the data analysis, carried out the initial analyses, drafted the initial manuscript, and reviewed and revised the manuscript. WB conceptualized and designed the study and methods for data collection, collected data, and reviewed and revised the manuscript. LM and AF collected data, reviewed the final analysis, and reviewed and revised the manuscript. SW build the statistical models, carried out the final analyses and critically reviewed the manuscript for important intellectual content. MR was senior supervisor of the project, conceptualized and designed the study, coordinated and supervised data collection, and critically reviewed the manuscript for important intellectual content. All authors approved the final manuscript as submitted and agree to be accountable for all aspects of the work.

## Conflict of Interest

The authors declare that the research was conducted in the absence of any commercial or financial relationships that could be construed as a potential conflict of interest.

## References

[1] Wood NS Costeloe K Gibson AT Hennessy EM Marlow N Wilkinson AR. The EPICure study: growth and associated problems in children born at 25 weeks of gestational age or less. Arch Dis Child Fetal Neonatal Ed. (2003) 88:F492–500. 10.1136/fn.88.6.F492 14602697 PMC1763245

[2] de Kieviet JF Zoetebier L van Elburg RM Vermeulen RJ Oosterlaan J. Brain development of very preterm and very low-birthweight children in childhood and adolescence: a meta-analysis. Dev Med Child Neurol. (2012) 54:313–23. 10.1111/j.1469-8749.2011.04216.x 22283622

[3] Cheong JL Anderson PJ Roberts G Burnett AC Lee KJ Thompson DK. Contribution of brain size to IQ and educational underperformance in extremely preterm adolescents. PLoS ONE. (2013) 8:e77475. 10.1371/journal.pone.0077475 24130887 PMC3793949

[4] El Marroun H Zou R Leeuwenburg MF Steegers EAP Reiss IKM Muetzel RL. Association of gestational age at birth with brain morphometry. JAMA Pediatr. (2020) 174:1149–58. 10.1001/jamapediatrics.2020.2991 32955580 PMC7506610

[5] Cheong JL Hunt RW Anderson PJ Howard K Thompson DK Wang HX. Head growth in preterm infants: correlation with magnetic resonance imaging and neurodevelopmental outcome. Pediatrics. (2008) 121:e1534–40. 10.1542/peds.2007-2671 18519457

[6] Gale CR O'Callaghan FJ Bredow M Martyn CN Avon Longitudinal Study of P Children Study T. The influence of head growth in fetal life, infancy, and childhood on intelligence at the ages of 4 and 8 years. Pediatrics. (2006) 118:1486–92. 10.1542/peds.2005-2629 17015539

[7] Cheong JL Burnett AC Lee KJ Roberts G Thompson DK Wood SJ. Association between postnatal dexamethasone for treatment of bronchopulmonary dysplasia and brain volumes at adolescence in infants born very preterm. J Pediatr. (2014) 164:737–43 e1. 10.1016/j.jpeds.2013.10.083 24332820 PMC4029072

[8] Coviello C Keunen K Kersbergen KJ Groenendaal F Leemans A Peels B. Effects of early nutrition and growth on brain volumes, white matter microstructure, and neurodevelopmental outcome in preterm newborns. Pediatr Res. (2018) 83:102–10. 10.1038/pr.2017.227 28915232

[9] Coviello C Popple Martinez M Drovandi L Corsini I Leonardi V Lunardi C. Painful procedures can affect post-natal growth and neurodevelopment in preterm infants. Acta Paediatr. (2018) 107:784–90. 10.1111/apa.14222 29341252

[10] Broere-Brown ZA Baan E Schalekamp-Timmermans S Verburg BO Jaddoe VW Steegers EA. Sex-specific differences in fetal and infant growth patterns: a prospective population-based cohort study. Biol Sex Differ. (2016) 7:65. 10.1186/s13293-016-0119-1 27980713 PMC5135770

[11] Kawasaki Y Yoshida T Matsui M Hiraiwa A Inomata S Tamura K. Clinical factors that affect the relationship between head circumference and brain volume in very-low-birth-weight infants. J Neuroimaging. (2019) 29:104–10. 10.1111/jon.12558 30260528 PMC6689194

[12] Ifflaender S Rudiger M Koch A Burkhardt W. Three-dimensional digital capture of head size in neonates - a method evaluation. PLoS ONE. (2013) 8:e61274. 10.1371/journal.pone.0061274 23580107 PMC3620274

[13] Wang S Fan P Xiong D Yang P Zheng J Zhao D. Assessment of neonatal brain volume and growth at different postmenstrual ages by conventional MRI. Medicine (Baltimore). (2018) 97:e11633. 10.1097/MD.0000000000011633 30075544 PMC6081163

[14] Boucher MA Lippe S Dupont C Knoth IS Lopez G Shams R. Computer-aided lateral ventricular and brain volume measurements in 3D ultrasound for assessing growth trajectories in newborns and neonates. Phys Med Biol. (2018) 63:225012. 10.1088/1361-6560/aaea85 30418939

[15] Burkhardt W Schneider D Hahn G Konstantelos D Maas HG Rudiger M. Non-invasive estimation of brain-volume in infants. Early Hum Dev. (2019) 132:52–7. 10.1016/j.earlhumdev.2019.03.020 30986647

[16] Fenton TR Kim JH. A systematic review and meta-analysis to revise the Fenton growth chart for preterm infants. BMC Pediatr. (2013) 13:59. 10.1186/1471-2431-13-59 23601190 PMC3637477

[17] Stasinopoulos DMRRA. Generalized Additive Models for Location Scale and Shape (GAMLSS) in R. J Stat Softw. (2007) 23:1–46. 10.18637/jss.v023.i07

[18] Kaczkurkin AN Raznahan A Satterthwaite TD. Sex differences in the developing brain: insights from multimodal neuroimaging. Neuropsychopharmacology. (2019) 44:71–85. 10.1038/s41386-018-0111-z 29930385 PMC6235840

[19] Alur P. Sex differences in nutrition, growth, and metabolism in preterm infants. Front Pediatr. (2019)7:22. 10.3389/fped.2019.00022 30792973 PMC6374621

[20] Ifflaender S Rudiger M Konstantelos D Wahls K Burkhardt W. Prevalence of head deformities in preterm infants at term equivalent age. Early Hum Dev. (2013) 89:1041–7. 10.1016/j.earlhumdev.2013.08.011 24016482

[21] Cole TJ. Sample size and sample composition for constructing growth reference centiles. Stat Methods Med Res. (2020) 30:88–507. 10.1177/0962280220958438 33043801 PMC8008444

[22] Hack M Breslau N Weissman B Aram D Klein N Borawski E. Effect of very low birth weight and subnormal head size on cognitive abilities at school age. N Engl J Med. (1991) 325:231–7. 10.1056/NEJM199107253250403 2057024

[23] Ehrenkranz RA Dusick AM Vohr BR Wright LL Wrage LA Poole WK. Growth in the neonatal intensive care unit influences neurodevelopmental and growth outcomes of extremely low birth weight infants. Pediatrics. (2006) 117:1253–61. 10.1542/peds.2005-1368 16585322

