# Supplementary material for: Reference Charts for Neonatal Cranial Volume Based on 3D Laser Scanning to Monitor Head Growth
Source: Front Pediatr. 2021 May 28;9:654112. doi: 10.3389/fped.2021.654112 (PMC8192695; doi:10.3389/fped.2021.654112)
Supplement: Supplementary file 1 [file Data_Sheet_1.docx]

Supplementary Material

1. **Supplementary Tables**

**1.1 Supplementary Table 1A.** Percentile Reference Table for Cranial Volume by Gestational Age in Girls

**1.2 Supplementary Table 1B.** Percentile Reference Table for Cranial Volume by Gestational Age in Boys

**1.3 Supplementary Table 1C.** Percentile Reference Table for Cranial Volume by Birth Weight in Girls

**1.4 Supplementary Table 1D.** Percentile Reference Table for Cranial Volume by Birth Weight in Boys

**2.1 Supplementary Table 2A.** Distributional parameters for Cranial Volume by Gestational Age

**2.2 Supplementary Table 2B.** Distributional parameters for Cranial Volume by Birth Weight

1. **Supplementary Figures**

**2.1 Supplementary Figure 1.** Flowchart on selection of study sample.

- 1. **Supplementary Figure 2A-B.** Reference curve for cranial volume by birth weight for girls (A) and boys (B).
  2. **Supplementary Figure 3A-D.** Raw data (grey dots) for cranial volume by gestational age for girls (A) and boys (B) and by birthweight for girls (C) and boys (D).
  3. **Supplementary Figure 4A-D.** Confidence intervals for the percentiles for cranial volume by gestational age for girls (A) and boys (B) and by birthweight for girls (C) and boys (D).

1. **Supplementary Tables**

**1.1 Supplementary Table 1A.** Percentile Reference Table for Cranial Volume by Gestational Age in Girls

| **Gestational age (wks)** | **P 2** | **P 10** | **P 25** | **Median** | **P 75** | **P 90** | **P 98** |
| --- | --- | --- | --- | --- | --- | --- | --- |
| 34 | 227.7 | 256.7 | 279.5 | 304.8 | 330.1 | 352.8 | 381.8 |
| 34 1/7 | 230.8 | 259.8 | 282.6 | 307.9 | 333.2 | 355.9 | 384.9 |
| 34 2/7 | 233.9 | 262.9 | 285.7 | 311.0 | 336.3 | 359.0 | 388.0 |
| 34 3/7 | 237.0 | 266.0 | 288.8 | 314.1 | 339.4 | 362.1 | 391.1 |
| 34 4/7 | 240.1 | 269.1 | 291.8 | 317.2 | 342.5 | 365.2 | 394.2 |
| 34 5/7 | 243.2 | 272.2 | 294.9 | 320.2 | 345.5 | 368.3 | 397.3 |
| 34 6/7 | 246.3 | 275.3 | 298.0 | 323.3 | 348.6 | 371.4 | 400.4 |
| 35 | 249.4 | 278.4 | 301.1 | 326.4 | 351.7 | 374.5 | 403.5 |
| 35 1/7 | 252.5 | 281.4 | 304.2 | 329.5 | 354.8 | 377.6 | 406.6 |
| 35 2/7 | 255.6 | 284.5 | 307.3 | 332.6 | 357.9 | 380.7 | 409.6 |
| 35 3/7 | 258.7 | 287.6 | 310.4 | 335.7 | 361.0 | 383.8 | 412.7 |
| 35 4/7 | 261.7 | 290.7 | 313.5 | 338.8 | 364.1 | 386.9 | 415.8 |
| 35 5/7 | 264.8 | 293.8 | 316.6 | 341.9 | 367.2 | 389.9 | 418.9 |
| 35 6/7 | 267.9 | 296.9 | 319.7 | 345.0 | 370.3 | 393.0 | 422.0 |
| 36 | 271.0 | 300.0 | 322.7 | 348.0 | 373.3 | 396.1 | 425.1 |
| 36 1/7 | 274.1 | 303.0 | 325.8 | 351.1 | 376.4 | 399.2 | 428.2 |
| 36 2/7 | 277.2 | 306.1 | 328.9 | 354.2 | 379.5 | 402.3 | 431.2 |
| 36 3/7 | 280.2 | 309.2 | 332.0 | 357.3 | 382.6 | 405.4 | 434.3 |
| 36 4/7 | 283.3 | 312.3 | 335.1 | 360.4 | 385.7 | 408.4 | 437.4 |
| 36 5/7 | 286.4 | 315.3 | 338.1 | 363.4 | 388.7 | 411.5 | 440.5 |
| 36 6/7 | 289.4 | 318.4 | 341.2 | 366.5 | 391.8 | 414.5 | 443.5 |
| 37 | 292.5 | 321.4 | 344.2 | 369.5 | 394.8 | 417.6 | 446.5 |
| 37 1/7 | 295.5 | 324.4 | 347.2 | 372.5 | 397.8 | 420.6 | 449.5 |
| 37 2/7 | 298.5 | 327.4 | 350.2 | 375.5 | 400.8 | 423.6 | 452.5 |
| 37 3/7 | 301.4 | 330.4 | 353.2 | 378.5 | 403.8 | 426.5 | 455.5 |
| 37 4/7 | 304.4 | 333.3 | 356.1 | 381.4 | 406.7 | 429.5 | 458.4 |
| 37 5/7 | 307.3 | 336.2 | 359.0 | 384.3 | 409.6 | 432.4 | 461.4 |
| 37 6/7 | 310.2 | 339.1 | 361.9 | 387.2 | 412.5 | 435.3 | 464.2 |
| 38 | 313.0 | 342.0 | 364.8 | 390.1 | 415.4 | 438.1 | 467.1 |
| 38 1/7 | 315.8 | 344.8 | 367.6 | 392.9 | 418.2 | 440.9 | 469.9 |
| 38 2/7 | 318.6 | 347.6 | 370.4 | 395.7 | 421.0 | 443.7 | 472.7 |
| 38 3/7 | 321.4 | 350.3 | 373.1 | 398.4 | 423.7 | 446.5 | 475.4 |
| 38 4/7 | 324.1 | 353.0 | 375.8 | 401.1 | 426.4 | 449.2 | 478.2 |
| 38 5/7 | 326.7 | 355.7 | 378.5 | 403.8 | 429.1 | 451.8 | 480.8 |
| 38 6/7 | 329.3 | 358.3 | 381.1 | 406.4 | 431.7 | 454.5 | 483.4 |
| 39 | 331.9 | 360.9 | 383.7 | 409.0 | 434.3 | 457.0 | 486.0 |
| 39 1/7 | 334.4 | 363.4 | 386.2 | 411.5 | 436.8 | 459.5 | 488.5 |
| 39 2/7 | 336.9 | 365.9 | 388.6 | 413.9 | 439.2 | 462.0 | 491.0 |
| 39 3/7 | 339.3 | 368.3 | 391.0 | 416.3 | 441.6 | 464.4 | 493.4 |
| 39 4/7 | 341.7 | 370.6 | 393.4 | 418.7 | 444.0 | 466.8 | 495.7 |
| 39 5/7 | 344.0 | 372.9 | 395.7 | 421.0 | 446.3 | 469.1 | 498.0 |
| 39 6/7 | 346.2 | 375.2 | 398.0 | 423.3 | 448.6 | 471.3 | 500.3 |
| 40 | 348.5 | 377.4 | 400.2 | 425.5 | 450.8 | 473.6 | 502.5 |
| 40 1/7 | 350.7 | 379.6 | 402.4 | 427.7 | 453.0 | 475.8 | 504.7 |
| 40 2/7 | 352.8 | 381.8 | 404.6 | 429.9 | 455.2 | 477.9 | 506.9 |
| 40 3/7 | 355.0 | 383.9 | 406.7 | 432.0 | 457.3 | 480.1 | 509.1 |
| 40 4/7 | 357.1 | 386.1 | 408.8 | 434.1 | 459.4 | 482.2 | 511.2 |
| 40 5/7 | 359.2 | 388.2 | 410.9 | 436.2 | 461.5 | 484.3 | 513.3 |
| 40 6/7 | 361.3 | 390.3 | 413.0 | 438.3 | 463.6 | 486.4 | 515.4 |
| 41 | 363.4 | 392.3 | 415.1 | 440.4 | 465.7 | 488.5 | 517.4 |
| 41 1/7 | 365.4 | 394.4 | 417.2 | 442.5 | 467.8 | 490.5 | 519.5 |
| 41 2/7 | 367.5 | 396.4 | 419.2 | 444.5 | 469.8 | 492.6 | 521.5 |
| 41 3/7 | 369.5 | 398.5 | 421.2 | 446.5 | 471.8 | 494.6 | 523.6 |
| 41 4/7 | 371.5 | 400.5 | 423.3 | 448.6 | 473.9 | 496.7 | 525.6 |
| 41 5/7 | 373.6 | 402.5 | 425.3 | 450.6 | 475.9 | 498.7 | 527.7 |
| 41 6/7 | 375.6 | 404.6 | 427.3 | 452.6 | 478.0 | 500.7 | 529.7 |
| 42 | 377.6 | 406.6 | 429.4 | 454.7 | 480.0 | 502.8 | 531.7 |

Shown are cranial volume values (ml) for different percentiles by gestational age (weeks). GA: gestational age; P 2: 2nd percentile; P 10: 10th percentile; etc. Tables with SD-scores, instead of percentiles, are available upon request to the first author.

- 1. **Supplementary Table 1B.** Percentile Reference Table for Cranial Volume by Gestational Age in Boys

| **Gestational age (wks)** | **P 2** | **P 10** | **P 25** | **Median** | **P 75** | **P 90** | **P 98** |
| --- | --- | --- | --- | --- | --- | --- | --- |
| 34 | 251.6 | 282.3 | 306.4 | 333.1 | 359.9 | 383.9 | 414.6 |
| 34 1/7 | 254.7 | 285.3 | 309.4 | 336.2 | 362.9 | 387.0 | 417.6 |
| 34 2/7 | 257.8 | 288.4 | 312.5 | 339.2 | 366.0 | 390.1 | 420.7 |
| 34 3/7 | 260.9 | 291.5 | 315.6 | 342.3 | 369.1 | 393.1 | 423.8 |
| 34 4/7 | 263.9 | 294.5 | 318.6 | 345.4 | 372.1 | 396.2 | 426.8 |
| 34 5/7 | 267.0 | 297.6 | 321.7 | 348.4 | 375.2 | 399.3 | 429.9 |
| 34 6/7 | 270.0 | 300.7 | 324.8 | 351.5 | 378.3 | 402.3 | 433.0 |
| 35 | 273.1 | 303.7 | 327.8 | 354.6 | 381.3 | 405.4 | 436.0 |
| 35 1/7 | 276.2 | 306.8 | 330.9 | 357.6 | 384.4 | 408.5 | 439.1 |
| 35 2/7 | 279.2 | 309.8 | 333.9 | 360.7 | 387.4 | 411.5 | 442.1 |
| 35 3/7 | 282.2 | 312.9 | 337.0 | 363.7 | 390.5 | 414.5 | 445.2 |
| 35 4/7 | 285.3 | 315.9 | 340.0 | 366.7 | 393.5 | 417.6 | 448.2 |
| 35 5/7 | 288.3 | 318.9 | 343.0 | 369.8 | 396.5 | 420.6 | 451.2 |
| 35 6/7 | 291.3 | 321.9 | 346.0 | 372.8 | 399.5 | 423.6 | 454.2 |
| 36 | 294.3 | 324.9 | 349.0 | 375.8 | 402.5 | 426.6 | 457.2 |
| 36 1/7 | 297.3 | 327.9 | 352.0 | 378.8 | 405.5 | 429.6 | 460.2 |
| 36 2/7 | 300.3 | 330.9 | 355.0 | 381.7 | 408.5 | 432.6 | 463.2 |
| 36 3/7 | 303.2 | 333.9 | 357.9 | 384.7 | 411.4 | 435.5 | 466.2 |
| 36 4/7 | 306.2 | 336.8 | 360.9 | 387.6 | 414.4 | 438.5 | 469.1 |
| 36 5/7 | 309.1 | 339.7 | 363.8 | 390.5 | 417.3 | 441.4 | 472.0 |
| 36 6/7 | 312.0 | 342.6 | 366.7 | 393.4 | 420.2 | 444.3 | 474.9 |
| 37 | 314.9 | 345.5 | 369.6 | 396.3 | 423.1 | 447.2 | 477.8 |
| 37 1/7 | 317.7 | 348.3 | 372.4 | 399.2 | 425.9 | 450.0 | 480.6 |
| 37 2/7 | 320.6 | 351.2 | 375.3 | 402.0 | 428.8 | 452.8 | 483.5 |
| 37 3/7 | 323.4 | 354.0 | 378.1 | 404.8 | 431.6 | 455.7 | 486.3 |
| 37 4/7 | 326.1 | 356.8 | 380.9 | 407.6 | 434.4 | 458.4 | 489.1 |
| 37 5/7 | 328.9 | 359.5 | 383.6 | 410.4 | 437.1 | 461.2 | 491.8 |
| 37 6/7 | 331.6 | 362.3 | 386.4 | 413.1 | 439.9 | 463.9 | 494.6 |
| 38 | 334.4 | 365.0 | 389.1 | 415.8 | 442.6 | 466.7 | 497.3 |
| 38 1/7 | 337.1 | 367.7 | 391.8 | 418.5 | 445.3 | 469.4 | 500.0 |
| 38 2/7 | 339.7 | 370.4 | 394.4 | 421.2 | 447.9 | 472.0 | 502.7 |
| 38 3/7 | 342.4 | 373.0 | 397.1 | 423.8 | 450.6 | 474.7 | 505.3 |
| 38 4/7 | 345.0 | 375.6 | 399.7 | 426.5 | 453.2 | 477.3 | 507.9 |
| 38 5/7 | 347.6 | 378.2 | 402.3 | 429.1 | 455.8 | 479.9 | 510.5 |
| 38 6/7 | 350.2 | 380.8 | 404.9 | 431.7 | 458.4 | 482.5 | 513.1 |
| 39 | 352.7 | 383.4 | 407.5 | 434.2 | 461.0 | 485.0 | 515.7 |
| 39 1/7 | 355.3 | 385.9 | 410.0 | 436.7 | 463.5 | 487.6 | 518.2 |
| 39 2/7 | 357.8 | 388.4 | 412.5 | 439.3 | 466.0 | 490.1 | 520.7 |
| 39 3/7 | 360.3 | 390.9 | 415.0 | 441.7 | 468.5 | 492.6 | 523.2 |
| 39 4/7 | 362.8 | 393.4 | 417.5 | 444.2 | 471.0 | 495.0 | 525.7 |
| 39 5/7 | 365.2 | 395.8 | 419.9 | 446.7 | 473.4 | 497.5 | 528.1 |
| 39 6/7 | 367.6 | 398.3 | 422.3 | 449.1 | 475.9 | 499.9 | 530.6 |
| 40 | 370.1 | 400.7 | 424.8 | 451.5 | 478.3 | 502.4 | 533.0 |
| 40 1/7 | 372.5 | 403.1 | 427.2 | 453.9 | 480.7 | 504.8 | 535.4 |
| 40 2/7 | 374.9 | 405.5 | 429.6 | 456.3 | 483.1 | 507.2 | 537.8 |
| 40 3/7 | 377.3 | 407.9 | 432.0 | 458.7 | 485.5 | 509.6 | 540.2 |
| 40 4/7 | 379.7 | 410.3 | 434.4 | 461.1 | 487.9 | 512.0 | 542.6 |
| 40 5/7 | 382.0 | 412.7 | 436.8 | 463.5 | 490.3 | 514.3 | 545.0 |
| 40 6/7 | 384.4 | 415.1 | 439.1 | 465.9 | 492.6 | 516.7 | 547.4 |
| 41 | 386.8 | 417.4 | 441.5 | 468.3 | 495.0 | 519.1 | 549.7 |
| 41 1/7 | 389.2 | 419.8 | 443.9 | 470.6 | 497.4 | 521.5 | 552.1 |
| 41 2/7 | 391.6 | 422.2 | 446.3 | 473.0 | 499.8 | 523.9 | 554.5 |
| 41 3/7 | 393.9 | 424.6 | 448.6 | 475.4 | 502.1 | 526.2 | 556.9 |
| 41 4/7 | 396.3 | 426.9 | 451.0 | 477.8 | 504.5 | 528.6 | 559.2 |
| 41 5/7 | 398.7 | 429.3 | 453.4 | 480.1 | 506.9 | 531.0 | 561.6 |
| 41 6/7 | 401.0 | 431.7 | 455.7 | 482.5 | 509.3 | 533.3 | 564.0 |
| 42 | 403.4 | 434.0 | 458.1 | 484.9 | 511.6 | 535.7 | 566.3 |

Shown are cranial volume values (ml) for different percentiles by gestational age (weeks). GA: gestational age; P 2: 2nd percentile; P 10: 10th percentile; etc. Tables with SD-scores, instead of percentiles, are available upon request to the first author.

- 1. **Supplementary Table 1C.** Percentile Reference Table for Cranial Volume by Birth Weight in Girls

| **Birthweight (g)** | **P 2** | **P 10** | **P 25** | **Median** | **P 75** | **P 90** | **P 98** |
| --- | --- | --- | --- | --- | --- | --- | --- |
| 1,800 | 236.0 | 258.9 | 277.0 | 297.0 | 317.0 | 335.1 | 358.0 |
| 1,900 | 244.5 | 267.4 | 285.5 | 305.5 | 325.5 | 343.6 | 366.5 |
| 2,000 | 253.0 | 275.9 | 294.0 | 314.0 | 334.0 | 352.1 | 375.0 |
| 2,100 | 261.5 | 284.4 | 302.4 | 322.5 | 342.5 | 360.5 | 383.5 |
| 2,200 | 269.9 | 292.8 | 310.9 | 330.9 | 350.9 | 369.0 | 391.9 |
| 2,300 | 278.3 | 301.2 | 319.3 | 339.3 | 359.3 | 377.4 | 400.3 |
| 2,400 | 286.7 | 309.6 | 327.6 | 347.7 | 367.7 | 385.7 | 408.7 |
| 2,500 | 294.9 | 317.9 | 335.9 | 355.9 | 376.0 | 394.0 | 416.9 |
| 2,600 | 303.1 | 326.1 | 344.1 | 364.1 | 384.2 | 402.2 | 425.1 |
| 2,700 | 311.2 | 334.2 | 352.2 | 372.2 | 392.3 | 410.3 | 433.2 |
| 2,800 | 319.2 | 342.1 | 360.1 | 380.2 | 400.2 | 418.2 | 441.2 |
| 2,900 | 326.9 | 349.9 | 367.9 | 387.9 | 408.0 | 426.0 | 448.9 |
| 3,000 | 334.5 | 357.5 | 375.5 | 395.5 | 415.5 | 433.6 | 456.5 |
| 3,100 | 341.9 | 364.9 | 382.9 | 402.9 | 423.0 | 441.0 | 463.9 |
| 3,200 | 349.2 | 372.1 | 390.1 | 410.2 | 430.2 | 448.2 | 471.2 |
| 3,300 | 356.3 | 379.2 | 397.3 | 417.3 | 437.3 | 455.4 | 478.3 |
| 3,400 | 363.3 | 386.3 | 404.3 | 424.3 | 444.4 | 462.4 | 485.3 |
| 3,500 | 370.3 | 393.2 | 411.3 | 431.3 | 451.3 | 469.3 | 492.3 |
| 3,600 | 377.2 | 400.2 | 418.2 | 438.2 | 458.3 | 476.3 | 499.2 |
| 3,700 | 384.2 | 407.1 | 425.1 | 445.2 | 465.2 | 483.2 | 506.2 |
| 3,800 | 391.1 | 414.0 | 432.1 | 452.1 | 472.1 | 490.2 | 513.1 |
| 3,900 | 398.1 | 421.0 | 439.0 | 459.1 | 479.1 | 497.1 | 520.1 |
| 4,000 | 405.0 | 428.0 | 446.0 | 466.0 | 486.1 | 504.1 | 527.0 |
| 4,100 | 412.0 | 435.0 | 453.0 | 473.0 | 493.1 | 511.1 | 534.0 |
| 4,200 | 419.0 | 441.9 | 460.0 | 480.0 | 500.0 | 518.1 | 541.0 |
| 4,300 | 426.0 | 448.9 | 467.0 | 487.0 | 507.0 | 525.1 | 548.0 |
| 4,400 | 433.0 | 455.9 | 474.0 | 494.0 | 514.0 | 532.0 | 555.0 |
| 4,500 | 440.0 | 462.9 | 480.9 | 501.0 | 521.0 | 539.0 | 562.0 |

Shown are cranial volume values (ml) for different percentiles by birth weight (g). BW: birth weight; P 2: 2nd percentile; P 10: 10th percentile; etc. Tables with SD-scores, instead of percentiles, are available upon request to the first author.

**1.4 Supplementary Table 1D.** Percentile Reference Table for Cranial Volume by Birth Weight in Boys

| **Birthweight (g)** | **P 2** | **P 10** | **P 25** | **Median** | **P 75** | **P 90** | **P 98** |
| --- | --- | --- | --- | --- | --- | --- | --- |
| 1,800 | 234.7 | 253.6 | 268.4 | 284.8 | 301.3 | 316.1 | 334.9 |
| 1,900 | 244.7 | 263.8 | 278.8 | 295.5 | 312.1 | 327.1 | 346.2 |
| 2,000 | 254.7 | 274.0 | 289.2 | 306.1 | 323.0 | 338.2 | 357.6 |
| 2,100 | 264.7 | 284.3 | 299.7 | 316.8 | 333.9 | 349.3 | 368.9 |
| 2,200 | 274.6 | 294.5 | 310.1 | 327.5 | 344.8 | 360.4 | 380.3 |
| 2,300 | 284.5 | 304.7 | 320.5 | 338.1 | 355.7 | 371.5 | 391.7 |
| 2,400 | 294.4 | 314.9 | 330.9 | 348.7 | 366.6 | 382.6 | 403.0 |
| 2,500 | 304.3 | 325.0 | 341.2 | 359.3 | 377.4 | 393.6 | 414.3 |
| 2,600 | 314.0 | 335.0 | 351.5 | 369.8 | 388.1 | 404.6 | 425.6 |
| 2,700 | 323.5 | 344.8 | 361.5 | 380.1 | 398.6 | 415.4 | 436.6 |
| 2,800 | 332.8 | 354.4 | 371.3 | 390.1 | 408.9 | 425.9 | 447.4 |
| 2,900 | 341.7 | 363.5 | 380.7 | 399.8 | 418.9 | 436.0 | 457.9 |
| 3,000 | 350.1 | 372.3 | 389.7 | 409.0 | 428.3 | 445.7 | 467.8 |
| 3,100 | 358.0 | 380.5 | 398.1 | 417.7 | 437.3 | 454.9 | 477.3 |
| 3,200 | 365.4 | 388.1 | 406.0 | 425.9 | 445.7 | 463.6 | 486.3 |
| 3,300 | 372.3 | 395.4 | 413.5 | 433.6 | 453.7 | 471.9 | 494.9 |
| 3,400 | 379.0 | 402.3 | 420.7 | 441.1 | 461.5 | 479.8 | 503.2 |
| 3,500 | 385.3 | 409.0 | 427.6 | 448.3 | 469.0 | 487.6 | 511.3 |
| 3,600 | 391.5 | 415.5 | 434.4 | 455.3 | 476.3 | 495.1 | 519.1 |
| 3,700 | 397.5 | 421.8 | 440.9 | 462.1 | 483.4 | 502.5 | 526.8 |
| 3,800 | 403.2 | 427.8 | 447.2 | 468.7 | 490.2 | 509.6 | 534.3 |
| 3,900 | 408.7 | 433.7 | 453.3 | 475.1 | 496.9 | 516.6 | 541.6 |
| 4,000 | 414.0 | 439.3 | 459.2 | 481.3 | 503.5 | 523.4 | 548.7 |
| 4,100 | 419.2 | 444.9 | 465.0 | 487.4 | 509.9 | 530.0 | 555.7 |
| 4,200 | 424.2 | 450.2 | 470.7 | 493.4 | 516.1 | 536.6 | 562.6 |
| 4,300 | 429.2 | 455.5 | 476.2 | 499.3 | 522.3 | 543.0 | 569.4 |
| 4,400 | 434.0 | 460.7 | 481.7 | 505.0 | 528.4 | 549.4 | 576.1 |
| 4,500 | 438.7 | 465.8 | 487.1 | 510.7 | 534.4 | 555.7 | 582.7 |

Shown are cranial volume values (ml) for different percentiles by birth weight (g). BW: birth weight; P 2: 2^nd^ percentile; P 10: 10^th^ percentile; etc. Tables with SD-scores, instead of percentiles, are available upon request to the first author.

**2.1 Supplementary Table 2A.** Distributional parameters for Cranial Volume by Gestational Age

|  | **Girls** |  | **Boys** |  |
| --- | --- | --- | --- | --- |
| **Gestational age (wks)** | **mu** | **sigma** | **mu** | **sigma** |
| 34 | 304.7536 | 37.51223 | 333.1053 | 39.66541 |
| 34 1/7 | 307.8542 | 37.51223 | 336.1760 | 39.66541 |
| 34 2/7 | 310.9541 | 37.51223 | 339.2460 | 39.66541 |
| 34 3/7 | 314.0529 | 37.51223 | 342.3149 | 39.66541 |
| 34 4/7 | 317.1501 | 37.51223 | 345.3822 | 39.66541 |
| 34 5/7 | 320.2454 | 37.51223 | 348.4471 | 39.66541 |
| 34 6/7 | 323.3385 | 37.51223 | 351.5091 | 39.66541 |
| 35 | 326.4294 | 37.51223 | 354.5671 | 39.66541 |
| 35 1/7 | 329.5186 | 37.51223 | 357.6203 | 39.66541 |
| 35 2/7 | 332.6064 | 37.51223 | 360.6676 | 39.66541 |
| 35 3/7 | 335.6933 | 37.51223 | 363.7078 | 39.66541 |
| 35 4/7 | 338.7796 | 37.51223 | 366.7399 | 39.66541 |
| 35 5/7 | 341.8658 | 37.51223 | 369.7627 | 39.66541 |
| 35 6/7 | 344.9518 | 37.51223 | 372.7747 | 39.66541 |
| 36 | 348.0373 | 37.51223 | 375.7748 | 39.66541 |
| 36 1/7 | 351.1220 | 37.51223 | 378.7614 | 39.66541 |
| 36 2/7 | 354.2043 | 37.51223 | 381.7333 | 39.66541 |
| 36 3/7 | 357.2821 | 37.51223 | 384.6894 | 39.66541 |
| 36 4/7 | 360.3532 | 37.51223 | 387.6282 | 39.66541 |
| 36 5/7 | 363.4143 | 37.51223 | 390.5483 | 39.66541 |
| 36 6/7 | 366.4620 | 37.51223 | 393.4484 | 39.66541 |
| 37 | 369.4929 | 37.51223 | 396.3270 | 39.66541 |
| 37 1/7 | 372.5043 | 37.51223 | 399.1831 | 39.66541 |
| 37 2/7 | 375.4941 | 37.51223 | 402.0156 | 39.66541 |
| 37 3/7 | 378.4600 | 37.51223 | 404.8241 | 39.66541 |
| 37 4/7 | 381.4005 | 37.51223 | 407.6090 | 39.66541 |
| 37 5/7 | 384.3141 | 37.51223 | 410.3709 | 39.66541 |
| 37 6/7 | 387.1993 | 37.51223 | 413.1101 | 39.66541 |
| 38 | 390.0537 | 37.51223 | 415.8269 | 39.66541 |
| 38 1/7 | 392.8750 | 37.51223 | 418.5214 | 39.66541 |
| 38 2/7 | 395.6607 | 37.51223 | 421.1937 | 39.66541 |
| 38 3/7 | 398.4079 | 37.51223 | 423.8432 | 39.66541 |
| 38 4/7 | 401.1137 | 37.51223 | 426.4698 | 39.66541 |
| 38 5/7 | 403.7752 | 37.51223 | 429.0732 | 39.66541 |
| 38 6/7 | 406.3896 | 37.51223 | 431.6534 | 39.66541 |
| 39 | 408.9539 | 37.51223 | 434.2105 | 39.66541 |
| 39 1/7 | 411.4661 | 37.51223 | 436.7448 | 39.66541 |
| 39 2/7 | 413.9264 | 37.51223 | 439.2567 | 39.66541 |
| 39 3/7 | 416.3352 | 37.51223 | 441.7467 | 39.66541 |
| 39 4/7 | 418.6940 | 37.51223 | 444.2165 | 39.66541 |
| 39 5/7 | 421.0063 | 37.51223 | 446.6680 | 39.66541 |
| 39 6/7 | 423.2761 | 37.51223 | 449.1034 | 39.66541 |
| 40 | 425.5074 | 37.51223 | 451.5252 | 39.66541 |
| 40 1/7 | 427.7048 | 37.51223 | 453.9359 | 39.66541 |
| 40 2/7 | 429.8732 | 37.51223 | 456.3380 | 39.66541 |
| 40 3/7 | 432.0167 | 37.51223 | 458.7333 | 39.66541 |
| 40 4/7 | 434.1382 | 37.51223 | 461.1234 | 39.66541 |
| 40 5/7 | 436.2403 | 37.51223 | 463.5094 | 39.66541 |
| 40 6/7 | 438.3257 | 37.51223 | 465.8919 | 39.66541 |
| 41 | 440.3969 | 37.51223 | 468.2713 | 39.66541 |
| 41 1/7 | 442.4560 | 37.51223 | 470.6479 | 39.66541 |
| 41 2/7 | 444.5056 | 37.51223 | 473.0221 | 39.66541 |
| 41 3/7 | 446.5478 | 37.51223 | 475.3942 | 39.66541 |
| 41 4/7 | 448.5849 | 37.51223 | 477.7647 | 39.66541 |
| 41 5/7 | 450.6186 | 37.51223 | 480.1338 | 39.66541 |
| 41 6/7 | 452.6499 | 37.51223 | 482.5019 | 39.66541 |
| 42 | 454.6797 | 37.51223 | 484.8692 | 39.66541 |

At each value of the gestational age the distribution of the cranial volume of girls and boys was estimated to have normal distribution with mean equal to mu and standard deviation equal to sigma.

**2.2 Supplementary Table 2B.** Distributional parameters for Cranial Volume by Birth Weight

|  | **Girls** |  | **Boys** |  |
| --- | --- | --- | --- | --- |
| **Birthweight (g)** | **mu** | **sigma** | **mu** | **sigma** |
| 1,800 | 296.9905 | 29.70000 | 284.8066 | 24.38543 |
| 1,900 | 305.5053 | 29.70000 | 295.4725 | 24.71580 |
| 2,000 | 313.9994 | 29.70000 | 306.1379 | 25.05064 |
| 2,100 | 322.4680 | 29.70000 | 316.7996 | 25.39002 |
| 2,200 | 330.9051 | 29.70000 | 327.4558 | 25.73400 |
| 2,300 | 339.3039 | 29.70000 | 338.1041 | 26.08264 |
| 2,400 | 347.6545 | 29.70000 | 348.7328 | 26.43600 |
| 2,500 | 355.9402 | 29.70000 | 359.3101 | 26.79415 |
| 2,600 | 364.1377 | 29.70000 | 369.7835 | 27.15715 |
| 2,700 | 372.2198 | 29.70000 | 380.0792 | 27.52507 |
| 2,800 | 380.1573 | 29.70000 | 390.1120 | 27.89797 |
| 2,900 | 387.9263 | 29.70000 | 399.7843 | 28.27593 |
| 3,000 | 395.5122 | 29.70000 | 408.9902 | 28.65900 |
| 3,100 | 402.9215 | 29.70000 | 417.6769 | 29.04727 |
| 3,200 | 410.1736 | 29.70000 | 425.8569 | 29.44079 |
| 3,300 | 417.2970 | 29.70000 | 433.6159 | 29.83965 |
| 3,400 | 424.3219 | 29.70000 | 441.0672 | 30.24391 |
| 3,500 | 431.2826 | 29.70000 | 448.2964 | 30.65365 |
| 3,600 | 438.2178 | 29.70000 | 455.3223 | 31.06894 |
| 3,700 | 445.1540 | 29.70000 | 462.1323 | 31.48985 |
| 3,800 | 452.1047 | 29.70000 | 468.7213 | 31.91647 |
| 3,900 | 459.0690 | 29.70000 | 475.1146 | 32.34887 |
| 4,000 | 466.0418 | 29.70000 | 481.3463 | 32.78712 |
| 4,100 | 473.0188 | 29.70000 | 487.4421 | 33.23131 |
| 4,200 | 480.0012 | 29.70000 | 493.4117 | 33.68152 |
| 4,300 | 486.9897 | 29.70000 | 499.2650 | 34.13783 |
| 4,400 | 493.9834 | 29.70000 | 505.0221 | 34.60032 |
| 4,500 | 500.9792 | 29.70000 | 510.7154 | 35.06908 |

At each value of the birthweight (increasing by steps of 100g) the distribution of the cranial volume of girls and boys was estimated to have normal distribution with mean equal to mu and standard deviation equal to sigma.

1. **Supplementary Figures**

**
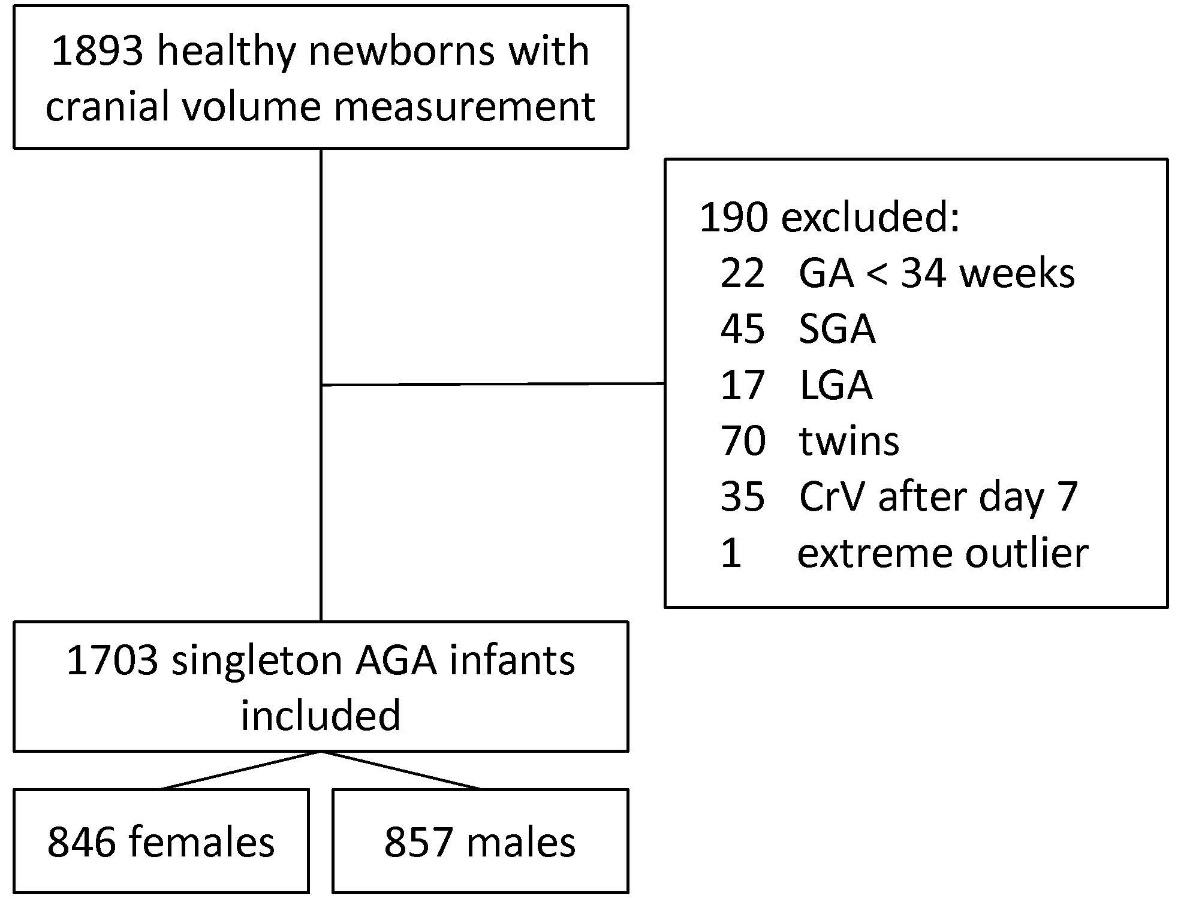
**

**2.1 Supplementary Figure 1.** Flowchart on selection of study sample.

N: number; GA: gestational age; SGA: small for gestational age; LGA: large for gestational age; AGA: appropriate for gestational age; CrV: cranial volume measurement

**
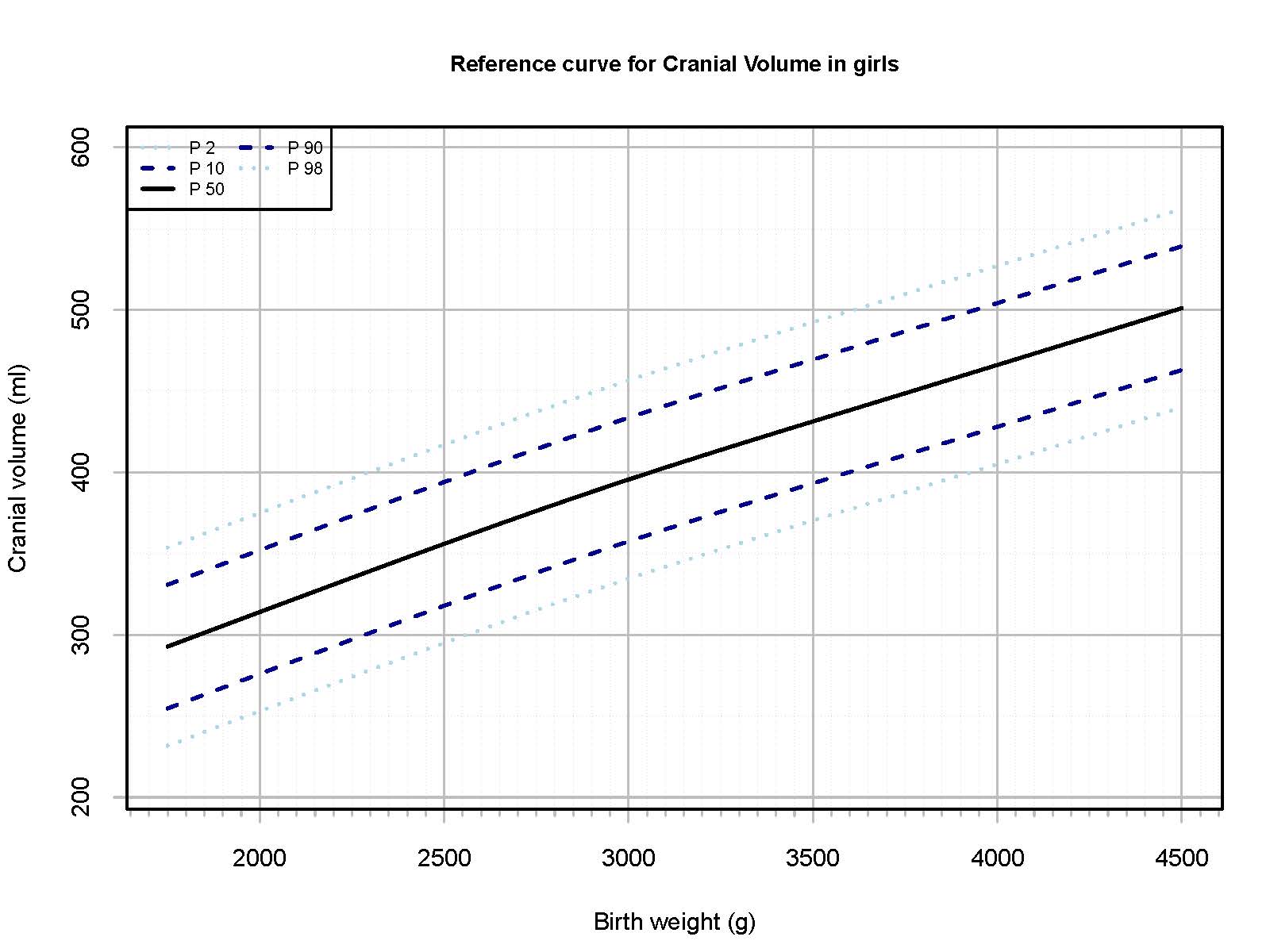
A.**


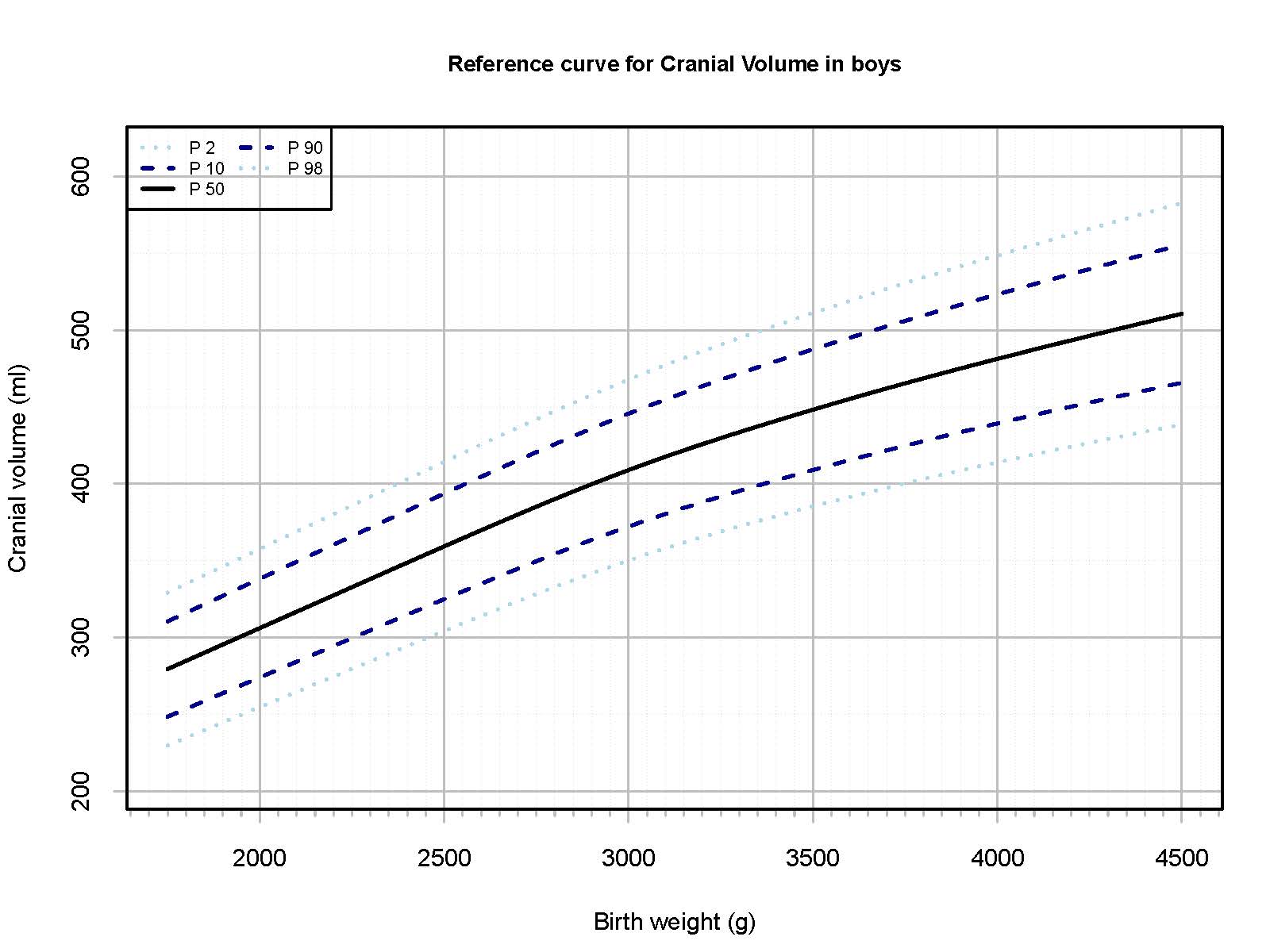


**B.**

- 1. **Supplementary Figure 2A-B.** Reference curve for cranial volume by birth weight for girls (A) and boys (B).

**A. B.**


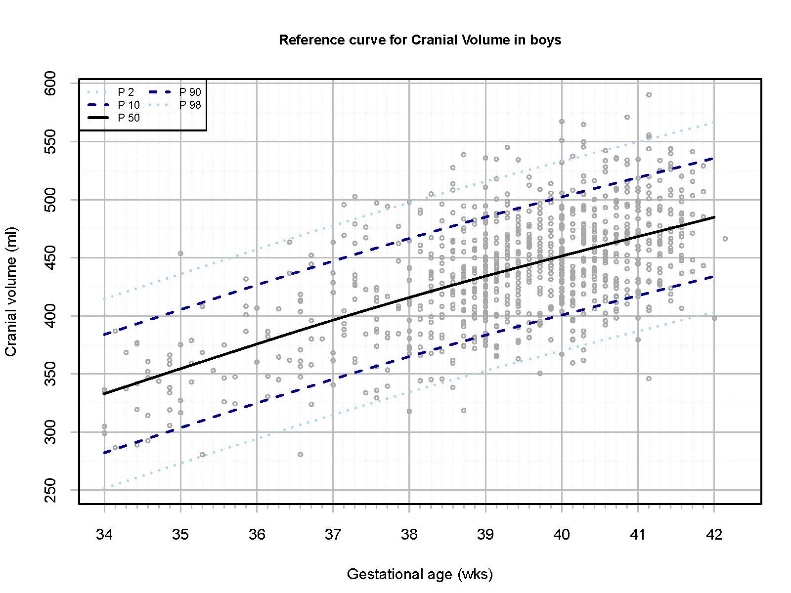
**
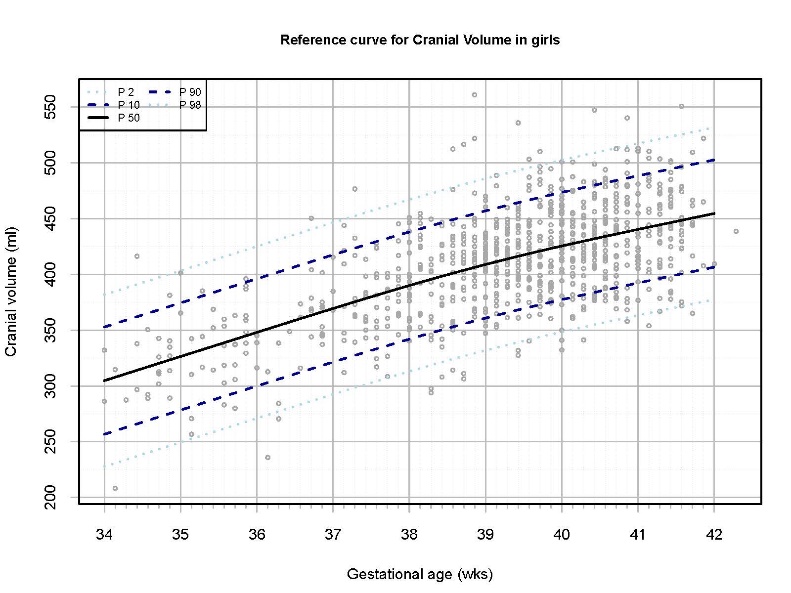
**

**B.**

**C.**


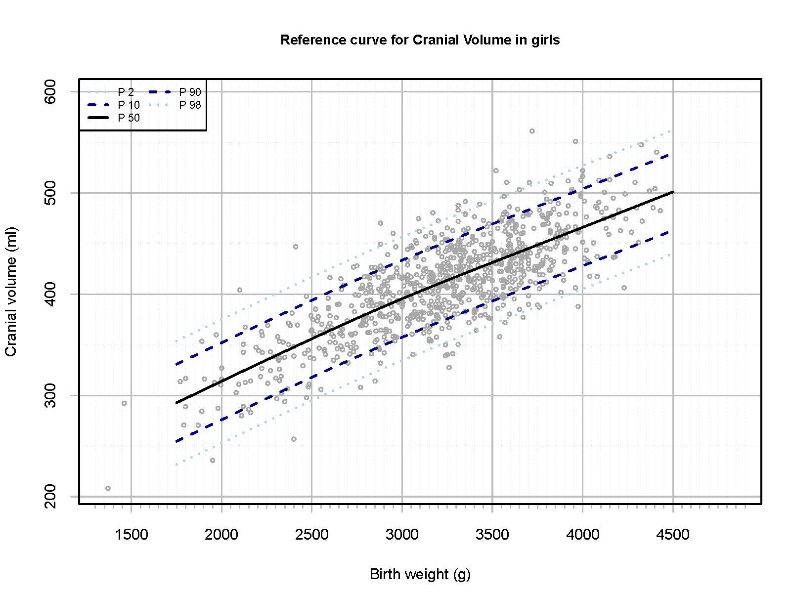
**C. D.**

**
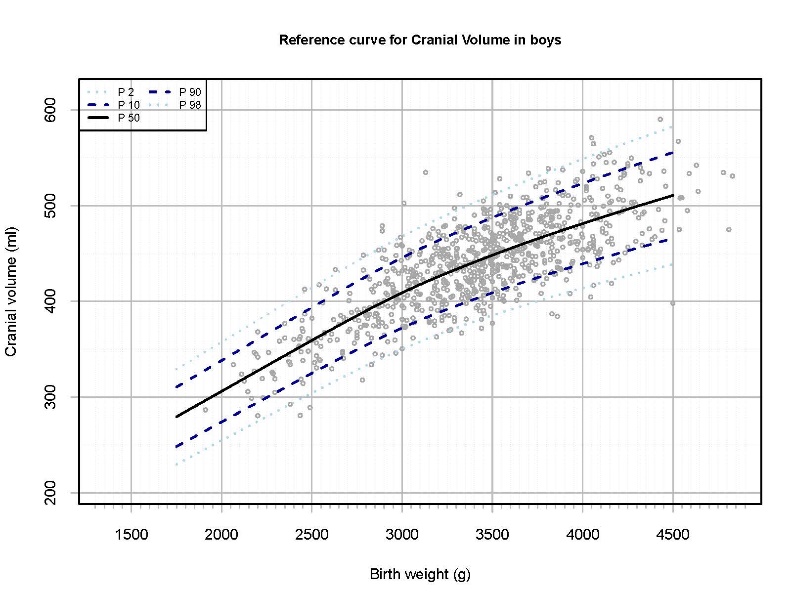
**

- 1. **Supplementary Figure 3A-D.** Raw data (grey dots) for cranial volume by gestational age for girls (A) and boys (B) and by birthweight for girls (C) and boys (D).

1. **B.**


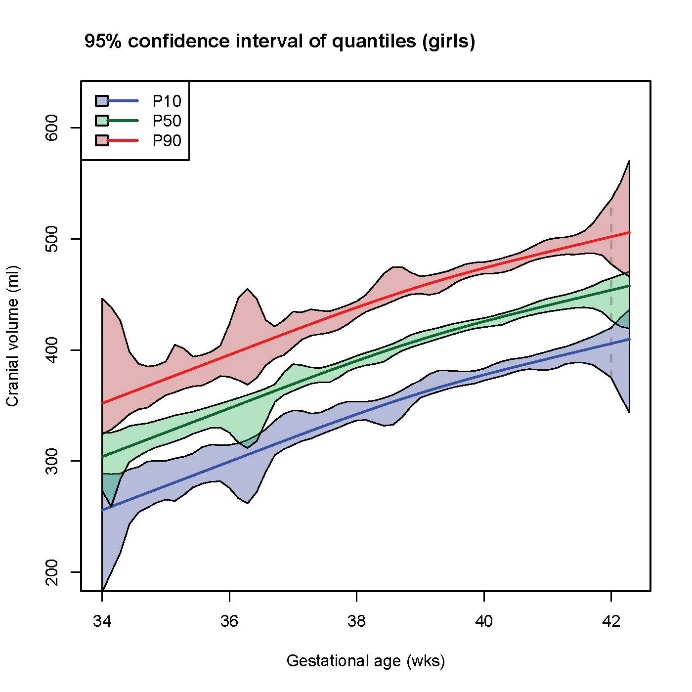

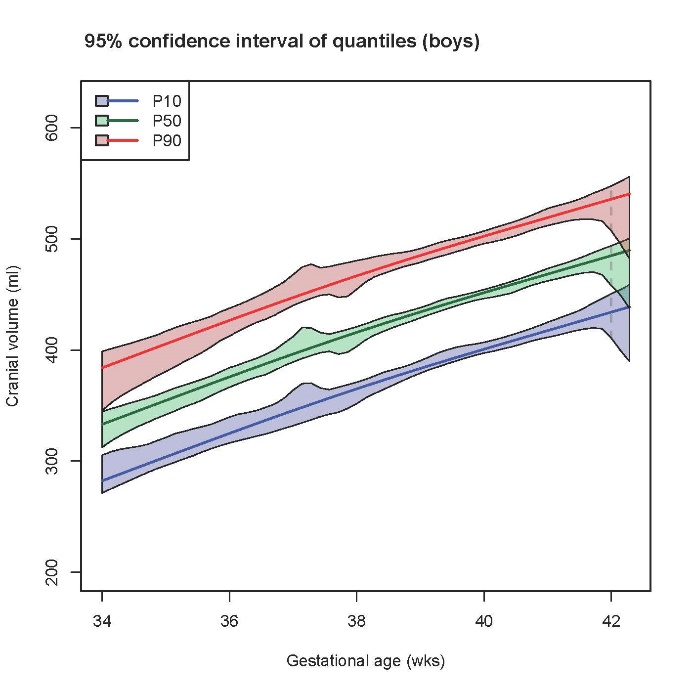


**C. D.**


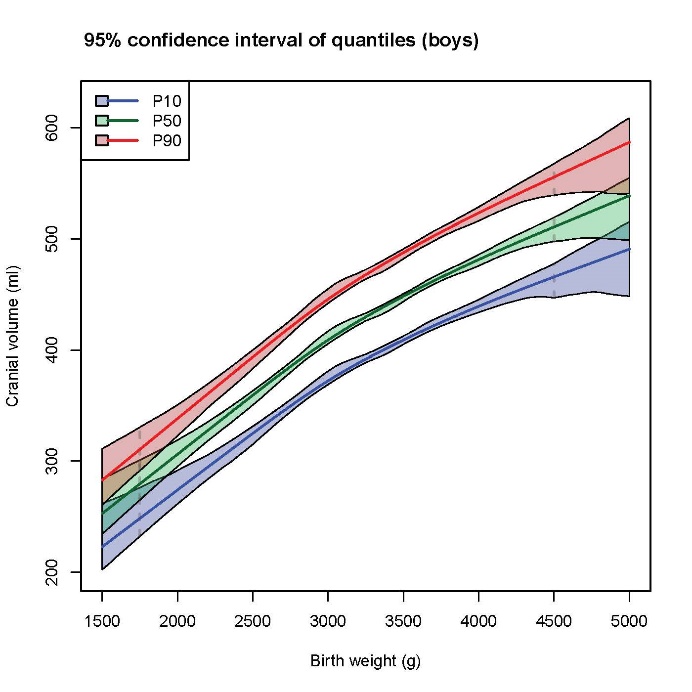
**
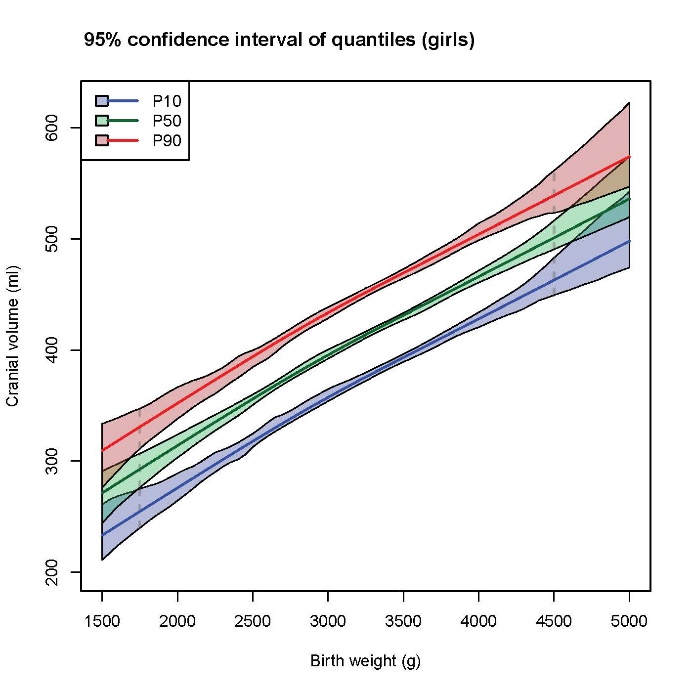
**

- 1. **Supplementary Figure 4A-D.** Confidence intervals for the percentiles for cranial volume by gestational age for girls (A) and boys (B) and by birthweight for girls (C) and boys (D). Grey dotted line indicates boundaries of X-axis in the final graphs.
